# Perspectives on Alzheimer’s Disease Treatment Based on Counteracting Oxidative Stress

**DOI:** 10.3390/biom15091345

**Published:** 2025-09-19

**Authors:** Rafał Bilski, Stanisław Dąbkowski, Igor Kozieł, Michał Kozicki, Anna Małachowska, Mikołaj Przygocki, Oliwia Tyska

**Affiliations:** 1 Department of Medical Biology and Biochemistry, Faculty of Medicine, Ludwik Rydygier Collegium Medicum in Bydgoszcz, Nicolaus Copernicus University in Toruń, 24 Karłowicza St., 85-092 Bydgoszcz, Poland; 2 Students Research Club of Medical Biology, Department of Medical Biology and Biochemistry, Faculty of Medicine, Ludwik Rydygier Collegium Medicum in Bydgoszcz, Nicolaus Copernicus University in Toruń, 87-100 Torun, Poland330755@stud.umk.pl (I.K.); 330932@stud.umk.pl (O.T.)

**Keywords:** Alzheimer disease, oxidative stress, antioxidants, therapeutic strategies, neurodegeneration

## Abstract

Alzheimer’s disease (AD) is a progressive neurodegenerative disorder and one of the most pressing global health challenges. Increasing evidence highlights oxidative stress as a key factor in its pathogenesis, contributing to amyloid-β accumulation, tau hyperphosphorylation, neuroinflammation, and mitochondrial dysfunction. Oxidative stress markers, detected in the bodily fluids of AD patients, are considered promising diagnostic and prognostic tools. Despite extensive research, currently available therapies remain largely symptomatic, which emphasizes the need to develop novel, disease-modifying strategies. The aim of this review is to summarize current knowledge on the role of oxidative stress in the pathogenesis of AD and to evaluate therapeutic approaches aimed at its reduction. We discuss molecular mechanisms linking reactive oxygen species to neurodegeneration and present pharmacological strategies such as monoamine oxidase inhibitors and multifunctional agents, as well as natural antioxidants, dietary interventions, and novel therapeutic technologies. We pay particular attention to their efficacy, limitations, and translational challenges. A more profound understanding of oxidative stress-related mechanisms may facilitate the development of combined antioxidant, anti-inflammatory, and neuroprotective approaches, offering new perspectives for delaying disease progression and improving patient outcomes.

## 1. Introduction

Alzheimer’s disease (AD) is a progressive, neurodegenerative disorder of growing social and medical importance. It is estimated that by the mid-21st century, the number of patients worldwide will exceed 150 million, placing AD at the center of public health and biomedical research. This disease, which most often develops in the elderly, is characterized by a slow decline in cognitive function, memory and language impairments, and, in later stages, a complete loss of independence [1]. The pathogenesis of AD is complex and multifactorial. Classic pathogenetic pathways include β-amyloid (Aβ) deposition in the form of senile plaques, hyperphosphorylation of tau protein leading to the formation of neurofibrillary tangles, chronic inflammation, and oxidative stress—a process that is both a cause and a consequence of the molecular abnormalities observed in AD [2]. Oxidative stress has traditionally been defined as an imbalance between the production of reactive oxygen species (ROS) and the antioxidant capacity of the cell. However, this classical definition is now considered outdated. According to the current understanding, oxidative stress should be viewed as a disturbance of redox signaling and control and/or molecular damage resulting from deviations in the cellular redox environment [3,4].

Modern redox biology emphasizes the concept of redox homeodynamics, which highlights the dynamic and adaptive nature of redox regulation rather than a static balance. Within this framework, a distinction is made between oxidative eustress, denoting physiological redox signaling required for normal cellular functions (e.g., H_2_O_2_-mediated cysteine oxidation), and oxidative distress, which reflects pathological oxidative imbalance leading to damage of proteins, lipids, and nucleic acids. Moreover, the notion of redox signaling and redox sensing provides a more integrative perspective, where thiol-based redox switches in proteins coordinate processes such as proliferation, metabolism, and apoptosis [5].

In the context of Alzheimer’s disease, this refined view suggests that impaired redox signaling—rather than simply ROS overproduction—contributes to amyloid-β aggregation, tau hyperphosphorylation, mitochondrial dysfunction, and neuroinflammation. Oxidative stress affects various stages of AD pathogenesis—it increases Aβ deposition, influences tau protein aggregation and promotes neuroinflammation, and mitochondrial dysfunction. Importantly, numerous studies have demonstrated the presence of oxidative stress markers in the body fluids of AD patients, such as malondialdehyde (MDA), 8-hydroxy-2′-deoxyguanosine (8-OHdG), and F2-isoprostanes, making them potential diagnostic and prognostic biomarkers. Natural defense systems, such as antioxidant enzymes like superoxide dismutase (SOD), catalase (CAT), glutathione peroxidase (GPx), and non-enzymatic compounds like glutathione (GSH), ascorbic acid, and alpha-lipoic acid, play a crucial role in neutralizing ROS. In AD, their activity is impaired, resulting in loss of redox homeostasis and increased neuronal degeneration [6,7]. Despite intensive research on disease-modifying drugs, current therapies are primarily based on symptom relief. Therefore, therapeutic approaches that aim to inhibit oxidative stress are receiving increasing attention. These include synthetic drugs—such as monoamine oxidase inhibitors (selegiline, rasagiline, ladostigil) [8,9]—and natural plant compounds (resveratrol, curcumin, EGCG, propolis, and vitamins C and E) [10,11], as well as dietary interventions and modern therapeutic technologies (nanoparticles, mitochondrial and gene therapies, immunotherapy) [12]. Examples of the latter include nanoparticle-based drug delivery systems designed to enhance brain penetration and antioxidant bioavailability [13], mitochondria-targeted agents that stabilize bioenergetics and reduce ROS production [14], gene therapy approaches aimed at reinforcing endogenous antioxidant pathways such as Nrf2 signaling [15], and immunotherapy strategies that indirectly alleviate oxidative stress by reducing amyloid-β burden and neuroinflammation [16].

The aim of this paper is to discuss the current state of knowledge regarding the role of oxidative stress in the pathogenesis of AD and to review therapeutic strategies aimed at reducing it. The molecular mechanisms involved in ROS will be discussed, as well as the efficacy and limitations of selected pharmacological and dietary approaches. Finally, the paper will address the challenges and prospects for further development of antioxidant therapies in the context of the complex pathophysiology of AD.

## 2. The Impact of Oxidative Stress on the Pathophysiology of Alzheimer’s Disease

**Figure 1:**
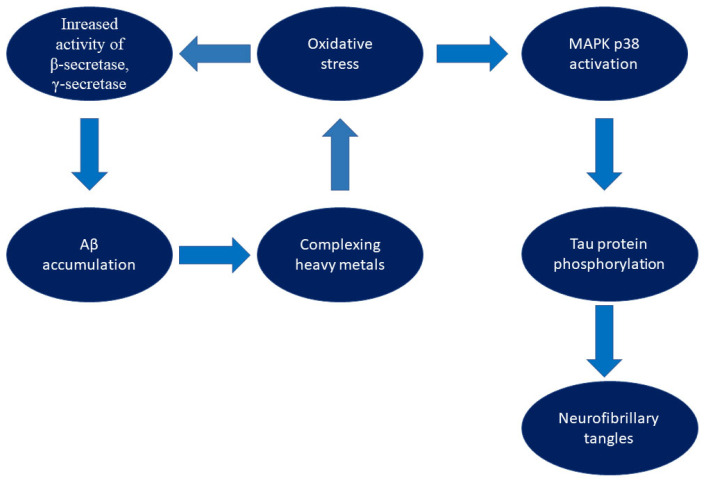
Schematic representation of oxidative stress-mediated pathways leading to amyloid-β accumulation and tau pathology in Alzheimer’s disease.

Oxidative stress plays a key role in the pathogenesis of AD, acting as both a consequence and a trigger for neurodegenerative processes. The CNS is highly sensitive to redox imbalances due to high oxygen metabolism, an abundance of unsaturated fatty acids, and limited neuronal regeneration. Endogenous antioxidant mechanisms cannot neutralize the excessive ROS production observed in AD [17,18]. Scientific literature highlights the link between oxidative stress and the accumulation of pathological beta-amyloid (Aβ) and hyperphosphorylated tau protein—two key markers of the disease. Aβ production is initiated by proteolytic cleavage of amyloid precursor protein (APP) by β- and γ-secretase, leading to the formation of toxic forms of Aβ, particularly Aβ42. Oxidative stress contributes to the increased activity of these enzymes, which enhances the deposition of Aβ in the form of amyloid plaques [19,20,21]. In turn, the presence of Aβ increases the production of ROS, particularly through the complexation of heavy metals such as copper and iron, which triggers Fenton reactions and leads to the formation of toxic hydroxyl radicals. This creates a vicious cycle: oxidative stress promotes the formation of Aβ, which in turn increases oxidative stress, contributing to the progression of neurodegeneration [22,23]. Hyperphosphorylated tau protein, another pathological marker of AD, also demonstrates a strong association with oxidative stress [7]. In conditions of excess ROS, kinases such as p38 MAPK are activated, which phosphorylate tau protein, destabilizing microtubules and leading to the formation of neurofibrillary tangles. These cytoskeletal disturbances promote neuronal cell death and loss of synaptic function [24,25]. Figure 1 illustrates the aforementioned pathomechanisms.

Another relevant factor linking oxidative stress and neurodegeneration in AD is the cellular prion protein (PrPC). Under physiological conditions, PrPC is involved in neuroprotection, metal ion homeostasis, and antioxidant defense, partly by modulating superoxide dismutase (SOD) activity and regulating redox balance. However, misfolded or oxidatively modified forms of PrPC lose these protective properties and may even exacerbate oxidative stress and amyloid-β toxicity. Studies suggest that PrPC can act as a binding partner for Aβ oligomers, contributing to synaptic dysfunction, while oxidative stress further destabilizes its physiological conformation, creating a vicious cycle that promotes neurodegeneration [26]. Thus, alterations in prion protein function should be considered an important component of the redox imbalance observed in Alzheimer’s disease. Mitochondria—the main source of ROS—also undergo dysfunction, which intensifies oxidative damage and reduces the level of ATP, essential for neuronal survival and function [27,28]. Abnormalities in the functioning of glial cells, particularly astrocytes, which under physiological conditions are involved in Aβ clearance and maintenance of the blood–brain barrier, are also exacerbated by oxidative stress [29]. Damage to the blood–brain barrier exacerbates the CNS’s exposure to toxic environmental factors and intensifies the inflammatory response [30]. One of the key defense mechanisms of neural cells against oxidative stress is the activation of the transcription factor Nrf2 (nuclear factor erythroid 2–related factor 2). Nrf2 is responsible for regulating over 200 genes involved in the antioxidant response, including those encoding enzymes such as SOD, CAT, GPx, heme oxygenase-1 (HO-1), and quinone oxidoreductase (NQO1). These enzymes neutralize ROS, protecting cells from damage [31]. Under physiological conditions, Nrf2 is bound by the protein Keap1, which directs it for degradation. However, under the influence of oxidative stress, Keap1 undergoes modifications, allowing Nrf2 to be released and translocate to the cell nucleus, where it activates defense genes [32]. In AD, this mechanism is disrupted. Increased Keap1 activity blocks Nrf2 activation, preventing the initiation of the cellular antioxidant response. As a result, nerve cells are more susceptible to damage caused by oxidative stress.

Disturbances in redox homeostasis are observed already at the stage of mild cognitive impairment (MCI), indicating that oxidative stress is one of the earliest factors in the pathogenesis of AD [27]. Decreased GSH levels, decreased activity of antioxidant enzymes (e.g., SOD, CAT), and reduced production of melatonin—an endogenous antioxidant—are observed in AD patients and may constitute a target for therapeutic strategies [33]. Table 1 provides a summary of changes in antioxidant and oxidative stress marker levels in AD patients.

In summary, oxidative stress plays a central role in the initiation and progression of AD, affecting Aβ production and aggregation, tau hyperphosphorylation, mitochondrial dysfunction, and glial cell function. A thorough understanding of these mechanisms is essential for developing effective therapies aimed at restoring redox balance in the CNS and delaying the neurodegenerative process in AD.

## 3. AD Therapies—A Review of Current Research and Therapeutic Approaches

Current pharmacological therapies for AD include acetylcholinesterase inhibitors and glutamate receptor antagonists. Despite the prevalence of the disease, only four treatment options have been approved in the European Union: donepezil, galantamine, and rivastigmine (acetylcholinesterase inhibitors), and memantine (an N-methyl-D-aspartate (NMDA) receptor antagonist). These drugs only have a symptomatic effect—they do not address the cause of the disease, but they alleviate and delay the progression of neuropsychiatric symptoms, which contributes to improving patients’ quality of life [48,49]. Intensive research has been ongoing for many years to develop disease-modifying therapies (DMTs), but due to the complex pathophysiology of AD, most of them end in failure [50]. One approach currently being studied is combination therapy, which involves adding DMTs to standard treatment [51,52]. Examples of such substances include monoamine oxidase inhibitors, such as selegiline and rasagiline, which exhibit neuroprotective potential and regulate oxidative stress [9]. An alternative research direction is the use of multifunctional molecules, such as ladostigil, combining different mechanisms of action.

Monoamine oxidases are mitochondrial enzymes that catalyze the breakdown of biogenic amines such as dopamine, serotonin, and noradrenaline. Two isoforms of the enzyme are distinguished: MAO-A and MAO-B, which differ primarily in their location in the nervous system, specificity for the degraded substrates, and sensitivity to inhibitors. MAO-B is present primarily in astrocytes and is responsible for the oxidation of dopamine and phenylethylamine. To a lesser extent, it also metabolizes noradrenaline and serotonin, which are the main substrates for MAO-A [53,54]. Oxidation of neurotransmitters is crucial for the proper functioning of synapses, but this process involves the production of compounds with potentially neurotoxic effects: hydrogen peroxide, ammonia, and aldehydes (R-CH_2_-NH_2_ + O_2_ + H_2_O --> R-CHO + NH_3_ + H_2_O_2_) [55]. Therefore, increased MAO-B activity contributes to oxidative stress and, consequently, to the development of neurodegenerative diseases such as Parkinson’s disease and AD [56]. Selegiline [(-)deprenyl, (R)-N,2-dimethyl-N-2-propynylphenylethylamine] and rasagiline [N-propargyl-(1R)(+)aminoindane] are selective, irreversible MAO-B inhibitors. They are commonly used as adjunctive therapies in the treatment of Parkinson’s disease and major depressive disorders [57,58,59]. Their mechanism of action involves blocking the breakdown of dopamine, increasing its availability in the nervous system, which alleviates the symptoms resulting from its deficiency. Although MAO-B inhibitors are not new therapeutic agents and many of the pivotal studies were published several decades ago, we included them in this review for two reasons. First, they represent one of the earliest pharmacological strategies in which attenuation of oxidative stress was a recognized component of the mechanism of action: by preventing the breakdown of dopamine and related monoamines, MAO-B inhibition reduces the generation of hydrogen peroxide and aldehydes, thereby exerting an indirect antioxidant effect. Second, they provide an instructive example of the challenges in translating preclinical antioxidant and neuroprotective findings into clinical benefit. Preclinical and clinical studies confirm the effectiveness of selegiline in reducing oxidative stress, which translates into improved cognitive function in patients with AD. However, clinical studies indicate that the effects in humans are often more moderate. Animal models with pharmacologically induced AD symptoms showed improved spatial memory following selegiline administration [60]. Selegiline administration led to improved spatial memory in animal models with pharmacologically induced AD symptoms, improved cognitive function but did not affect the level of insoluble Aβ1-42 [61]. The last significant clinical trials conducted in the 1990s focused on using selegiline to treat AD. Over the years, interest in this drug has declined due to limited long-term efficacy [62,63]. Rasagiline also demonstrates beneficial effects in studies, but its action appears to be more advanced due to its stronger neuroprotective properties. This benefit is manifested by increased CAT activity, regulation of the apoptotic pathway (increased Bcl/Bax ratio), and the absence of the formation of potentially neurotoxic metabolites (selegiline is metabolized to amphetamine derivatives) [64]. Clinical trials that combine rasagiline with acetylcholinesterase inhibitors have demonstrated an increase in cerebral blood flow, indicating an improvement in neuronal function [65]. Furthermore, studies indicate possible beneficial properties of rasagiline independent of MAO-B inhibition, opening new possibilities for its use in combination therapy for AD [66]. An alternative avenue of research is the use of multifunctional molecules combining both monoamine oxidase and cholinesterase inhibitor activity. This includes the experimental drug ladostigil, which, thanks to its synergistic action, simultaneously exhibits neuroprotective, anti-inflammatory, and antidepressant properties. Preclinical studies have indicated that ladostigil can inhibit microglial activation [67]. Researchers have demonstrated the drug’s multifaceted effects in aging rat models, which include a reduction in the expression of proapoptotic genes and those encoding proinflammatory cytokines. Additionally, ladostigil inhibited the expression of axonal growth inhibitors and genes that disrupt mitochondrial function, and also protected against aging-related impairments in episodic and spatial memory [68]. These results suggest a potential protective effect of ladostigil against aging-related neurodegeneration. Despite its promising therapeutic potential, clinical trials to date have yielded mixed results, keeping ladostigil in the experimental phase. Currently, there are no new active clinical trials underway. The most recently completed phase II study in 2019 did not demonstrate a significant effect on cognitive improvement in patients with mild cognitive impairment (MCI), but a slower rate of total brain volume decline was observed compared to the placebo group [69].

Despite the widespread use of selegiline in the treatment of Parkinson’s disease and major depressive disorders, its safety remains controversial [58,59]. Selegiline administered in doses up to 10 mg daily exhibits selectivity only for MAO-B, but at higher doses, this selectivity disappears, resulting in additional inhibition of MAO-A—an enzyme that metabolizes, among other things, tyramine. Consequently, the so-called cheese effect may occur, involving the accumulation of tyramine in the body after consuming foods rich in this amine, such as ripened cheeses or fermented products. This effect leads to a rapid increase in blood pressure, the so-called hypertensive crisis [70]. Another controversial aspect of selegiline use is its chemical structure—this drug is a derivative of methamphetamine, so it is broken down in the body into L-amphetamine and L-methamphetamine [50]. Although the L-isomers are less psychoactive than their D-configuration counterparts, overuse of selegiline can lead to agitation and insomnia. Rasagiline, like selegiline, also raises some safety concerns regarding its long-term use. One potential risk is serotonin syndrome (SS), which may occur with concomitant use of rasagiline and serotonergic drugs. However, this complication is relatively rare. It was reported, for example, in a 77-year-old man who used rasagiline in combination with a serotonin reuptake inhibitor (ESCRI) (escitalopram, used as an antidepressant) [71]. The patient experienced hallucinations, agitation, and fever. Despite discontinuing the medication, he also developed kidney damage. SS has also been observed with the combination of rasagiline with duloxetine or linezolid [71,72]. Despite the above-mentioned cases, a 2014 study of 1504 people with Parkinson’s disease assessed the potential risk of SSc. 471 patients were taking rasagiline and antidepressants, 511 patients were taking rasagiline alone, and 525 patients were taking antidepressants and dopaminergic drugs other than rasagiline and selegiline. No symptoms of serotonin syndrome were observed in either group, suggesting that the risk of this complication is low [73]. The summary of MAO-B inhibitors in AD therapy is presented in Table 2. It should be noted that monoamine oxidase-B (MAO-B) inhibitors, although not classical antioxidants, were included in this section because their mechanism of action indirectly reduces oxidative stress by limiting the generation of hydrogen peroxide and other reactive by-products during neurotransmitter catabolism. Their neuroprotective potential in Alzheimer’s disease has been attributed not only to symptomatic effects on dopaminergic transmission but also to the modulation of apoptotic pathways, mitochondrial function, and oxidative stress. Nevertheless, these drugs are not novel agents—selegiline and rasagiline have been in clinical use for decades, and more recent developments such as ladostigil remain experimental. Therefore, they are discussed here primarily as examples of pharmacological strategies that target oxidative stress through indirect mechanisms rather than as new antioxidant compounds.

Beyond monoamine oxidase-B inhibitors, several emerging therapeutic strategies for Alzheimer’s disease (AD) have been proposed, reflecting the complex and multifactorial nature of the disorder. A growing body of research focuses on anti-amyloid and anti-tau therapies, including monoclonal antibodies such as aducanumab, lecanemab, and donanemab, which aim to reduce amyloid-β plaques, and experimental tau immunotherapies designed to limit tau aggregation and propagation [74,75]. Parallel efforts target neuroinflammation, where drugs modulating microglia (e.g., CSF1R inhibitors), astrocytes (e.g., STAT3 inhibitors), or systemic metabolic pathways (e.g., insulin sensitizers, GLP-1 receptor agonists) are under investigation [74,76]. Another promising direction involves neuroprotective agents and redox-modulating compounds such as NMDAR modulators, sodium benzoate, and mitochondrial stabilizers, which act on excitotoxicity and oxidative stress-related pathways [74]. Finally, novel technologies, including nanoparticle-based delivery systems, antisense oligonucleotides, gene therapy and proteolysis-targeting chimeras (PROTACs), represent innovative approaches for improving drug targeting and efficacy [75]. Collectively, these therapeutic avenues underscore that while classical agents such as MAO-B inhibitors provide historical insights into antioxidant strategies, the current landscape is shifting toward multimodal and disease-modifying interventions that integrate antioxidant, anti-inflammatory, and neuroprotective effects. Recent progress in the therapeutic strategies in Alzheimer disease is presented in Table 3.

## 4. Natural Compounds and Diet

Natural compounds with antioxidant properties are receiving increasing attention in the context of AD prevention and adjunctive therapy. In selecting natural compounds for this review, we focused on agents with documented antioxidant and neuroprotective potential supported by preclinical and/or clinical evidence. Priority was given to compounds that are able to cross the blood–brain barrier or indirectly modulate central nervous system redox homeostasis, have been consistently reported to influence key pathogenic pathways in Alzheimer’s disease, such as amyloid-β deposition, tau phosphorylation, mitochondrial dysfunction, or neuroinflammation, and represent substances with translational potential due to their availability as dietary components or nutraceuticals. Based on these criteria, we included polyphenols, vitamins, melatonin, sulfur-containing compounds, and carotenoids. These agents exemplify different classes of natural products that act through complementary mechanisms—from direct radical scavenging to modulation of redox-sensitive signaling pathways such as Nrf2/Keap1, SIRT1/FOXO, or PI3K/AKT. The most frequently studied include polyphenols such as resveratrol, curcumin, epigallocatechin gallate (EGCG), vitamins C and E, and propolis. These compounds, found primarily in plant-based diets, have the ability to neutralize free radicals. Studies indicate that polyphenols can activate the Nrf2 pathway, leading to increased expression of endogenous antioxidant enzymes such as heme oxygenase-1 (HO-1) and SOD [100]. Scientific data confirm that resveratrol supplementation improves spatial memory and increases the activity of antioxidant enzymes (SOD, GSH-Px, CAT) while simultaneously reducing malondialdehyde (MDA) levels—a marker of lipid peroxidation [101]. Meta-analyses indicate the ability of pure curcumin to reduce MDA concentrations and increase total antioxidant capacity (TAC). By scavenging reactive nitrogen and oxygen, regulating enzymes, and chelating metals, curcumin demonstrates its antioxidant properties [102]. Studies have also demonstrated that curcumin’s hydroxyl groups stabilize ROS, thereby preventing DNA damage [103]. Individual vitamins play a crucial role in preventing oxidative stress. Tocopherol and tocotrienol derivatives scavenge free radicals present in cell membranes, thus protecting them against lipid peroxidation. Vitamin E inhibits hydrogen peroxide production, thus demonstrating cytotoxicity inhibitory activity [104]. Research findings confirm that this vitamin exhibits protective effects against impaired water maze performance associated with treatment with a neurotoxin (AF64A) that stimulates oxidative stress in cholinergic neurons [105]. Vitamin C’s role is primarily in counteracting ROS. Ascorbic acid is released by glial cells in the CNS, participating in the regeneration of antioxidant enzymes such as GSH and CAT [106]. In the context of antioxidant mechanisms, it has also been established that vitamin C is responsible for scavenging oxygen and nitrogen radicals, donating hydrogen atoms to lipid radicals, quenching singlet oxygen, removing molecular oxygen, and regenerating tocopherol from its oxidized form. Ascorbic acid is considered an excellent electron donor due to its low reduction potential (282 mV) [107]. An important complement is the increased antioxidant capacity of vitamin C in combination with polyphenols [108]. Furthermore, antioxidant activity is attributed to the active ingredient in green tea—EGCG. This compound exhibits a protective effect against lipid peroxidation, protecting cells from factors that initiate this process, such as t-butyl peroxide, 6-hydroxydopamine, iron ions, UV radiation, hydrogen peroxide, and 3-hydroxykynurenine. In vivo studies have indicated that EGCG reduces the levels of thiobarbituric acid-reactive substances (TBARS). A reduction in other markers of oxidative damage, such as lipid hydroperoxides, 4-hydroxynonenal (4-HNE), and MDA, was also observed. Concomitantly, an increase in GPx activity and reduced GSH levels were observed, indicating that EGCG enhances endogenous antioxidant defenses [109]. EGCG may reduce the hyperphosphorylation of the tau protein and downregulate BACE1 and Aβ1-42 expression, enhancing the antioxidant system and cognitive functions in rats with AD [110]. In vitro studies confirm the strong antioxidant properties of the chemical compound propolis. Both propolis aqueous extract (PWE) and phenolic compounds isolated from it, such as fraxetin, apigenin, galangin, pinobanksin, and chrysin, demonstrated significant free radical scavenging and reducing properties (including in DPPH and FRAP tests) [111]. A randomized, double-blind, placebo-controlled clinical trial assessed the tolerability and efficacy of oral curcumin in patients with mild to moderate AD. Thirty-six individuals were enrolled and randomly assigned to one of three groups: placebo, 2 g, or 4 g of curcumin daily for 24 weeks. The analysis of the results did not reveal significant differences between groups in cognitive function (ADAS-Cog, MMSE), neuropsychiatric symptoms (NPI), ability to perform daily activities (ADCS-ADL), or biomarkers of neurodegeneration and oxidative stress (Aβ1-40, Aβ1-42, t-tau, p-tau181, F2-isoprostanes in cerebrospinal fluid). The preparation was generally well tolerated; three participants experienced gastrointestinal symptoms that led to study withdrawal. Despite the relative safety of the treatment, the main limitation was the very low bioavailability of the use of curcumin, as evidenced by trace concentrations of the active substance in plasma (mean 7.32 ng/mL). Additionally, the small number of participants and the limited duration of the study may affect the generalizability of the results. The collected data do not confirm the clinical efficacy of curcumin in the treatment of AD, indicating the need for further studies using preparations with better bioavailability and longer follow-up [112].

Rutin is a natural flavonoid widely found in plant-based diets, known for its antioxidant, anti-inflammatory, and neuroprotective properties. Particular attention has been paid to its ability to modulate the Keap1/Nrf2 pathway, which plays a key role in the cellular response to oxidative stress [113]. In silico studies have shown that rutin binds to the Keap1 protein through hydrogen bonding with key amino acid residues (e.g., Ser602, Asn382, Asn387), blocking its interaction with Nrf2 and enabling transcriptional activation of genes encoding antioxidant enzymes such as HO-1, NQO1, SOD, and GPx [114]. In a model of sporadic AD (ICV-STZ), rutin administration (25 mg/kg p.o.) for 21 days resulted in significant improvement in spatial memory and reduction in oxidative stress in the hippocampus. Researchers observed a reduction in TBARS and nitrite levels, along with an increase in GPx, GR, and catalase activity. Rutin also reduced the expression of proinflammatory mediators such as iNOS, COX-2, and IL-8 [115]. In the Tau-P301S transgenic model, rutin inhibited tau protein hyperphosphorylation and promoted its degradation, which was associated with increased PP2A phosphatase activity and decreased NF-κB pathway activity. The Morris Water Maze and Y-maze tests also revealed a reduction in microglial activation and improved cognitive function [116]. In summary, rutin exhibits multifaceted neuroprotective effects—activating the Nrf2 pathway, reducing oxidative stress and inflammation, inhibiting tau toxicity, and supporting cognitive function. Available preclinical studies provide consistent and promising data that justify further translational and clinical research on this compound in the context of AD.

Sulforaphane (SFN), a bioactive isothiocyanate found mainly in cruciferous vegetables (e.g., broccoli, Brussels sprouts, kale), has gained particular attention as a natural compound with neuroprotective potential in the context of AD. Due to its ability to activate the Nrf2–ARE transcriptional pathway, SFN induces the expression of numerous detoxification and antioxidant enzymes, such as SOD, CAT, and glutathione S-transferase (GST), which leads to a reduction in oxidative stress in neuronal cells [117,118]. In in vivo studies, conducted, among others, on 3xTg-AD transgenic mice, oral administration of sulforaphane at doses of 10–50 mg/kg for 8 weeks resulted in a significant reduction in the levels of Aβ amyloid and tau protein and its phosphorylated forms. An increase in the expression of chaperone proteins (heat shock proteins—HSP70), improved cognitive functions, and reduced spatial memory deficits were also observed [119,120]. The administration of SFN to PS1V97L mice also yielded similar effects, confirming the reproducibility of the results [121]. In cellular models (e.g., SH-SY5Y exposed to Aβ), sulforaphane demonstrated the ability to reduce Aβ accumulation and the level of ROS. This mechanism was mediated by a decrease in the expression of histone deacetylases (HDAC1 and HDAC3) and an increase in the expression of the p75NTR receptor, which plays a protective role against the neurotoxic effects of amyloid. Interestingly, the reduction in HDAC1/3 led to an increase in the level of acetylated histones H3 and H4, which promoted neuroprotective transcriptional changes [122]. Furthermore, in silico studies indicate that sulforaphane may regulate multiple molecular pathways relevant to AD, including those related to inflammation (TNF), apoptosis (BCL2), insulin resistance (INS), and nitric oxide biogenesis. SFN has the ability to cross the blood–brain barrier, is characterized by a favorable pharmacokinetic profile (no CYP450 inhibition, no transport via P-glycoprotein), and has immunomodulatory activity [123]. Based on the available preclinical data, sulforaphane appears to be a promising candidate for adjunctive therapy in AD, although large-scale clinical trials confirming its efficacy in humans are currently lacking.

Melatonin (N-acetyl-5-methoxytryptamine), a neurohormone synthesized primarily by the pineal gland, is known for its circadian rhythm-regulating effects. Recent years have emphasized its neuroprotective and antioxidant properties, especially in the context of AD. In the brain, melatonin acts as a free radical scavenger, a modulator of signaling pathways, and a regulator of inflammatory processes and neurogenesis [7]. Melatonin exhibits a broad spectrum of neuroprotective effects in AD, affecting both the fundamental molecular mechanisms and the functional manifestations of neurodegeneration. One of its key mechanisms of action is the modulation of APP metabolism. Melatonin promotes the non-amyloidogenic pathway by increasing the expression of α-secretase (ADAM10), leading to the formation of the neuroprotective fragment sAPPα. At the same time, it inhibits the activity of β-secretase (BACE1) and γ-secretase (PS1), thereby limiting the production of the neurotoxic peptide Aβ1-42 [124,125,126]. Melatonin also supports mitochondrial function by stabilizing cardiolipin and respiratory chain enzymes, which results in reduced ROS production and prevents the formation of pathological mitochondrial complexes [127]. Under oxidative stress, it reduces the expression of activated caspase-3 and the Bax/Bcl-2 ratio, thus preventing neuronal apoptosis [128,129]. In the context of sleep disorders, commonly found in AD, melatonin improves sleep quality, prolongs REM sleep, and reduces nocturnal awakenings, which translates into better cognitive functioning and reduced neuropsychiatric symptoms. This effect results, among others, from the stabilization of clock gene expression (BMAL1, PER2), which improves neuronal rhythmicity and synchronization of sleep centers [130,131]. Melatonin also has a beneficial effect on the blood–brain barrier (BBB), reducing its permeability and limiting the penetration of inflammatory mediators into the CNS. Additionally, it exhibits anti-inflammatory effects by inhibiting NADPH oxidase activity and supporting the production of anti-inflammatory cytokines, such as IL-10 and neurotrophins [132]. In the context of metabolic disorders, often coexisting with AD, melatonin regulates the PI3K/AKT/mTOR pathway, improves IGF-1 activity, and limits IRS-1 phosphorylation, which promotes improved glucose transport, increased ATP availability, and reduced oxidative stress in conditions of insulin resistance [133,134]. Melatonin also influences key transcriptional pathways, such as SIRT1/FOXO, Nrf2/ARE, Wnt/β-catenin, and MAPK/ERK, which supports neuronal differentiation, limits apoptosis, and stimulates neural tissue regeneration [127,135]. It also counteracts excitotoxicity by stabilizing calcium metabolism—it inhibits excessive activation of NMDA receptors, regulates calcium-dependent kinases (CaMKII, PKC), and influences calcium-binding proteins such as calreticulin [136]. In animal models with scopolamine-induced amnesia, melatonin improved cognitive functions, restored the activity of the cholinergic system in the hippocampus and medial septum, and increased the expression of key proteins: ChAT, CHT, VAChT, and the M1 receptor [137]. Finally, melatonin supports neurogenesis and synaptic plasticity—it increases the proliferation of progenitor cells and supports their differentiation and functional integration, acting through the Wnt, Notch, PI3K/AKT pathways and trophic factors (BDNF, NGF, CREB). Studies in models of aging and AD have shown that long-term melatonin supplementation limits neurodegeneration, improves cognitive function, reduces Aβ deposition and tau phosphorylation, and inhibits microglial activation and inflammatory cytokine expression [7,138]. Melatonin exhibits multifaceted neuroprotective effects in AD, including reduction in oxidative stress, modulation of amyloid metabolism, mitochondrial support, and improvement of cholinergic function. Its ability to simultaneously regulate multiple pathological pathways makes it a promising candidate for adjunctive therapy in AD.

N-acetylcysteine (NAC) is a cysteine derivative known for its antioxidant activity and ability to increase GSH levels, the main intracellular antioxidant. Its effectiveness in alleviating the symptoms of AD is the subject of intensive preclinical research. Animal models of AD have shown that NAC and its derivative, N-acetylcysteine amide (NACA), can limit oxidative stress, reduce β-amyloid deposits, improve cognitive function, and stabilize the integrity of the blood–brain barrier [139,140]. In a study using rats in which AD-type pathology was induced by infusion of the Aβ1-42 peptide into the lateral ventricles of the brain, the use of NACA (75 mg/kg) in both prophylactic and therapeutic models significantly improved scores on the Morris Water Maze test, indicating improved spatial learning and memory. Additionally, histopathological analysis revealed reduced Aβ deposits, reduced tau phosphorylation, improved neuronal morphology, and reduced astrocyte and microglial activation. Researchers also observed increased expression of synaptophysin, a protein crucial for synaptic integrity, and improved hippocampal neurogenesis [141]. In a separate study using 5xFAD transgenic mice with aggressive amyloidopathy, dietary NAC supplementation (600 mg/kg) for 4 weeks resulted in reduced levels of the oxidative marker 4-HNE in the brain and a reduction in Aβ40 amyloid concentration. Importantly, NAC improved blood–brain barrier permeability, increased P-gp transporter activity, and reduced inflammation associated with microangiopathy. Treatment also led to improved cognitive function, as assessed by the Y-maze test [140]. Despite abundant preclinical evidence, the use of NAC in AD therapy faces significant limitations. NAC is characterized by low oral bioavailability (<10%), which makes it difficult to achieve therapeutic concentrations in the CNS. Alternative forms, such as NACA, are being developed, which are characterized by better lipophilicity and a greater ability to cross the blood–brain barrier [139,141]. Clinical studies on NAC to date are limited and inconclusive. In a randomized trial in patients with mild cognitive impairment (MCI), the use of a supplement containing NAC (600 mg/day for 12 weeks), combined with other ingredients (including vitamin B, ALA), resulted in moderate improvement in cognitive scores (ADAS-Cog, MoCA), but does not allow for a clear assessment of the efficacy of NAC alone. The authors recommend further studies in larger populations and with clear monotherapy [142,143,144]. Additionally, significant challenges in assessing the clinical utility of NAC arise from the lack of standardized doses and duration of therapy in clinical trials to date, as well as limited data on its long-term safety in older adults. Some sources also point to potential interactions with psychotropic medications and variability in effects depending on the stage of the disease [145]. In summary, NAC remains an intriguing candidate for the treatment of AD, but further, better-designed clinical trials are necessary to confirm its efficacy and safety.

Astaxanthin (ASX), a natural carotenoid with potent antioxidant properties, shows promising potential as an adjunctive therapy in the treatment of AD. In vitro models have shown that ASX promotes the clearance of toxic forms of Aβ from the brain by activating autophagy and increasing the expression of membrane transporters ABCA1 and ABCG1 and the LRP1 receptor, which improves the clearance of amyloid deposits across the blood–brain barrier. Simultaneously, ASX downregulates mTOR protein levels and reduces S6 protein phosphorylation, which supports autophagy activation. From an immunomodulatory perspective, ASX reduces the secretion of proinflammatory cytokines and promotes microglial polarization toward the M2 phenotype, which is associated with anti-inflammatory effects [146]. In animal models of AD, astaxanthin administration improved spatial memory, reduced oxidative stress, and inhibited neuronal apoptosis. This effect was mediated, among others, by the SIRT1/PGC-1α signaling pathway, which regulates the level of ROS and mitochondrial function. Furthermore, astaxanthin decreased the level of proapoptotic enzymes (e.g., Bax) and increased the expression of Bcl-2, thus protecting neurons from cell death [147]. In models of scopolamine-induced neurodegeneration, ASX effectively inhibited acetylcholinesterase activity and attenuated the levels of phosphorylated tau protein via the Akt/pAkt pathway [148]. Studies on modern forms of administration indicate that the use of astaxanthin nanoemulsions can significantly improve its bioavailability and neuroprotective efficacy. In a streptozotocin-induced AD model, ASX nanoemulsion led to improved cognitive functions, reduced Aβ and phosphorylated tau levels, and increased acetylcholine concentration in the brain [149]. A literature review also indicates that synergistic use of astaxanthin with other antioxidants, such as SOD, may enhance the neuroprotective effect, counteract the accumulation of neurotoxic proteins, and support neurogenesis in the hippocampus [150]. Importantly, ASX as an adjunct therapy may affect not only oxidative stress and inflammation but also neuroplasticity and mitochondrial energy functions [151]. Clinically, although most available studies involve preclinical models, there are indications that ASX supplementation may improve cognitive function in older adults without causing significant adverse effects. Human studies have reported subjective and objective improvements in cognitive function following ASX supplementation, although more randomized controlled trials with larger samples are needed to confirm these observations [152]. Furthermore, ASX also influences the regulation of microRNA-124 expression and the activation of Nrf2, a key regulator of the antioxidant response in neurons. This effect suggests a possible impact of ASX on broader regulatory networks relevant to neuroprotection in AD [153]. In summary, astaxanthin exerts multifaceted neuroprotective effects by reducing oxidative stress, modulating inflammatory pathways, and improving blood–brain barrier function. Promising preclinical results suggest that ASX may be a valuable adjunct therapy in the treatment of AD, although further validation in clinical trials is required.

Selenium is an essential trace element that plays a fundamental role in the antioxidant defense system through the activity of selenoproteins such as glutathione peroxidases and thioredoxin reductases. In the context of AD, selenium and its derivatives have recently attracted considerable attention due to their pleiotropic biological effects. Preclinical studies demonstrate that selenium compounds not only mitigate oxidative stress but also modulate tau phosphorylation, inhibit amyloid-β aggregation, and suppress neuroinflammation [154]. Representative examples include ebselen, a glutathione peroxidase mimetic with neuroprotective activity comparable to donepezil; selenocysteine derivatives and selenofuranosides, which improved cognition and reduced neuropathology in AD mouse models; as well as sodium selenate, which attenuated tau hyperphosphorylation, albeit with reported dose-dependent neurotoxicity. More recently, selenium nanoparticles (SeNPs) have emerged as a particularly promising platform due to their high biocompatibility, ability to cross the blood–brain barrier, and reduced systemic toxicity compared to inorganic salts [155]. SeNPs display strong antioxidant and anti-inflammatory effects, disrupt amyloid-β fibrillization, and exert synergistic neuroprotection when combined with polyphenols such as resveratrol. Functionalized SeNPs, including probiotic-derived formulations, have further demonstrated modulation of the gut–brain axis and improved cognitive performance in AD models [156]. Despite these encouraging outcomes, selenium-based therapies remain largely at the preclinical stage, and challenges related to dosing, safety, and long-term effects need to be resolved before translation into clinical application. Nevertheless, the multifaceted mechanisms of selenium chemistry highlight its potential as a valuable adjunct in future antioxidant-based therapeutic strategies for AD.

**Figure 2:**
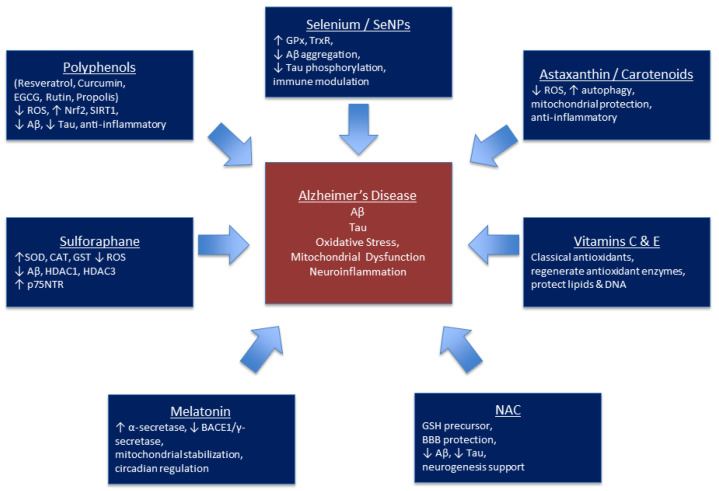
Schematic representation of the mechanisms of action of selected natural compounds and antioxidants in Alzheimer’s disease.

The MIND (Mediterranean-DASH Intervention for Neurodegenerative Delay) diet is also gaining increasing interest in the context of antioxidant therapy for AD. It combines elements of the Mediterranean and DASH diets, focusing on delaying neurodegeneration by addressing the mechanisms underlying dementia, such as oxidative stress [157]. The components of this diet contain compounds with documented antioxidant effects—green vegetables and berries are rich in polyphenols, carotenoids, and anthocyanins responsible for neutralizing ROS and limiting oxidative damage to DNA, lipids, and proteins [158,159]. Olive oil is a source of polyphenols and monounsaturated fatty acids, which stabilize cell membranes and enhance antioxidant defense [160]. Fish provide omega-3 fatty acids responsible for neutralizing ROS [161]. Studies indicate that a Mediterranean diet contributes to an increased Aβ42/40 ratio in cerebrospinal fluid and, in individuals with MCI, to a reduction in unfavorable changes in tau and amyloid biomarkers [162]. These results support the potential of the diet as a therapeutic strategy for preventing or delaying AD by reducing oxidative stress. Oxidative stress plays a key role in the pathogenesis of AD, and compounds with antioxidant properties represent an important area of research into neuroprotective strategies. Numerous natural substances, such as polyphenols (e.g., resveratrol, curcumin), vitamins C and E, melatonin, and plant compounds (EGCG, sulforaphane, rutin), have been shown to reduce ROS, regulate antioxidant pathways, and stabilize mitochondrial function. Consequently, they may limit Aβ deposition, inhibit tau protein phosphorylation, reduce neuroinflammatory cytokines, and improve cognitive function. An overview of selected mechanisms and the potential importance of antioxidants in the context of AD is presented in Table 4 and schematic overview of the main mechanisms of natural antioxidant compounds in the context of Alzheimer’s disease is presented in Figure 2.

Although not all of the neuroprotective compounds discussed in this section have reached advanced clinical testing, their therapeutic potential is supported by a growing body of evidence from both preclinical and clinical studies. Resveratrol has been investigated in several phase II clinical trials, demonstrating its ability to modulate cerebrospinal fluid biomarkers (e.g., Aβ40, tau) and exert mild cognitive benefits in patients with mild to moderate Alzheimer’s disease, despite limitations in bioavailability [163]. Vitamin C, while not tested in interventional trials, has shown a strong inverse association with Alzheimer’s-related mortality in a large epidemiological cohort (NHANES III), suggesting that adequate serum levels may confer protective effects [164]. Sulforaphane, although still in the preclinical phase, has demonstrated consistent neuroprotective effects across multiple animal models of AD, including reductions in amyloid and tau pathology, oxidative stress, and neuroinflammation [117]. Most notably, recent studies on rutin delivered via nitrogen-doped carbon dots (NCDs–rutin) revealed that a single 10 mg/kg dose produced comparable behavioral and molecular effects to a 30-day regimen of 50 mg/kg free rutin, indicating significantly enhanced bioavailability and therapeutic efficiency [165]. Staxanthin has been tested in four randomized controlled trials involving middle-aged and elderly individuals, showing improvements in memory, processing speed, and psychomotor performance, with no significant adverse effects [152]. Melatonin, although its clinical results are mixed, has demonstrated promising effects on sleep regulation, oxidative stress, tau phosphorylation, and neurogenesis in both preclinical and early clinical studies [166]. Collectively, these findings support the translational potential of natural compounds in AD therapy and highlight the importance of innovative delivery systems and circadian regulation in enhancing their clinical applicability. The summary of discussed therapeutic strategies is presented in Table 5.

A comparative analysis of antioxidant strategies in AD highlights significant differences in their translational potential. Natural polyphenols such as resveratrol and curcumin demonstrate pleiotropic neuroprotective effects but are hindered by poor bioavailability. Classical antioxidants like vitamins C and E are well studied but show inconsistent results in clinical settings. Melatonin offers an additional benefit through circadian rhythm regulation, though clinical outcomes remain variable. Selenium-based compounds, particularly selenium nanoparticles (SeNPs), represent a novel and promising line of research but remain mostly at the preclinical stage, with safety and dosing challenges to be addressed. MAO-B inhibitors provide a historical example of antioxidant-based therapy but are limited by safety and tolerability concerns. The most promising future direction lies in emerging multimodal strategies—including nanotechnology-based delivery, immunotherapy, and mitochondrial/gene-targeted approaches—which may allow for more effective interventions that combine antioxidant properties with broader neuroprotective mechanisms.

## 5. Conclusions

Oxidative stress plays a central role in the pathogenesis of Alzheimer’s disease, contributing to amyloid-β deposition, tau hyperphosphorylation, mitochondrial dysfunction, and neuroinflammation. Numerous antioxidant strategies have been proposed to counteract these processes; however, their translational success has been limited. A comparative analysis of available evidence highlights both opportunities and challenges. Natural polyphenols such as resveratrol and curcumin exhibit pleiotropic effects, including modulation of redox homeostasis, amyloid aggregation, and inflammatory signaling. Nevertheless, their therapeutic potential is constrained by poor bioavailability and rapid metabolism, underscoring the need for innovative delivery systems such as nanoparticles or prodrug formulations. Classical antioxidants, including vitamins C and E, have been extensively studied, but clinical outcomes remain inconsistent, suggesting that monotherapy with conventional radical scavengers is unlikely to be effective. Selenium-based compounds, particularly organic derivatives and selenium nanoparticles, are emerging as promising candidates with multi-target properties, ranging from antioxidant and anti-inflammatory actions to modulation of tau pathology. The future of antioxidant therapy in AD is likely to lie in multimodal and combinatorial strategies that integrate antioxidant, anti-inflammatory, and neuroprotective mechanisms. Emerging technologies—including nanoparticle-based delivery systems, mitochondrial-targeted drugs, gene therapy, and immunotherapeutic approaches—offer innovative avenues to overcome the shortcomings of conventional antioxidants. Rigorous clinical trials, improved biomarkers of redox imbalance, and personalized therapeutic strategies will be critical to advancing this field. Future progress in this area will also depend on the integration of in vitro and in vivo experimental models with computational approaches, which can accelerate the identification of novel antioxidant candidates and rational drug combinations. Although this aspect lies beyond the primary scope of the present review, it represents an important direction for future research in AD therapy. In summary, while traditional antioxidants have provided valuable mechanistic insights, novel approaches that combine redox regulation with broader disease-modifying actions hold the greatest potential for effective translation into Alzheimer’s disease therapy.

## 6. Limitations

Despite the broad scope and relevance of the issues discussed, this review paper has several significant limitations that should be considered when interpreting the data presented. First, a significant portion of the analyzed results come from preclinical studies, including animal models or in vitro experiments. Although these provide valuable information on the molecular mechanisms of oxidative stress in the pathogenesis of AD, their translation to the clinical setting remains limited. There are significant differences in pharmacokinetics, bioavailability, and the organization of neurodegenerative pathways between experimental models and the human central nervous system. Second, the effectiveness of many of the discussed antioxidant strategies in clinical trials remains equivocal. Some of the analyzed interventions are based on studies with small sample sizes, lack of an appropriate control group, limited observation time, or low bioavailability of the active substances used. These factors make it difficult to draw definitive conclusions about their effectiveness in clinical practice. Third, the complex and multifactorial nature of AD pathophysiology makes it difficult to clearly determine the role of oxidative stress as a primary etiological factor. Many pathological processes in AD—such as Aβ aggregation, mitochondrial dysfunction, and glial cell activation—interact with oxidative mechanisms, which can lead to difficulties in assessing causality. Furthermore, the review does not specifically address some new and promising therapeutic strategies, such as cell therapy, modulation of the gut microbiome, or targeted biological therapies, which may also modulate oxidative stress and influence the course of the disease. Finally, due to the dynamic development of AD research and the constant emergence of new scientific reports, the presented state of knowledge may change as clinical and molecular research progresses.

## Figures and Tables

**Table 1:** Changes in antioxidants and oxidative stress marker levels in AD patients.

Parameter	Levels in AD Patients	References
Total Antioxidant Status (TAS)	Decreased	[34,35]
MDA	Increased	[36,37,38,39,40]
8-OHdG	Increased	[41]
SOD	Decreased	[33,36,40]
GSH	Decreased	[33,42,43]
CAT	Decreased	[33]
	Increased	[39]
GPx	Decreased	[33,36,40,44]
Advanced oxidation protein products (AOPP)	Increased	[45]
Ischemia-modified albumin (IMA)	Increased	[45]
Ferric reducing antioxidant power (FRAP)	Decreased	[45]
Prooxidant–antioxidant balance (PAB)	Increased	[45]
Melatonin	Decreased	[46,47]

**Table 2:** Characteristics of selected MAO-B inhibitors in Alzheimer’s disease therapy.

Drug	Mechanism of Action	Effectiveness	Comments	References
Selegiline	Irreversible MAO-B inhibitor. Reduces dopamine breakdown and hydrogen peroxide production, lowering oxidative stress—neuroprotective effect.	Improvement of cognitive function in preclinical trials. Moderate efficacy in humans.	At doses above 10 mg/day may cause the “cheese effect”. Metabolized to amphetamine derivatives.	[59,60,61,62]
Rasagiline	Irreversible MAO-B inhibitor. In addition to reducing dopamine breakdown, it increases catalase activity.	Potentially more advanced effects than selegiline, beneficial outcomes in studies.	In combination with serotonin reuptake inhibitors may cause serotonin syndrome.	[63,64,65]
Ladostigil	Multifunctional drug combining cholinesterase and MAO-B inhibition. Exhibits anti-inflammatory, anti-apoptotic effects, reduces microglial activation and oxidative stress.	Mixed results in clinical studies.	Experimental drug.	[66,67,68]

**Table 3:** Emerging therapeutic strategies in Alzheimer’s disease.

Therapeutic Strategy	Mechanism of Action	Current Status	References
Anti-amyloid antibodies (Aducanumab, Lecanemab, Donanemab)	Promote clearance of amyloid-β plaques and prevent aggregation	FDA-approved/Phase 3 clinical trials	[75,77,78,79]
Tau-targeted therapies (immunotherapies, antisense oligonucleotides, aggregation inhibitors)	Reduce tau phosphorylation, aggregation, and propagation	Preclinical/Early clinical trials	[74,80,81,82]
Neuroinflammation modulators (CSF1R inhibitors, STAT3 inhibitors, GLP-1 receptor agonists)	Modulate microglial and astrocytic activity, suppress pro-inflammatory cascades	Preclinical/Ongoing clinical trials	[74,83,84,85,86,87,88]
Mitochondrial stabilizers and redox modulators (e.g., sodium benzoate, NMDAR modulators)	Counteract excitotoxicity, stabilize mitochondrial bioenergetics, reduce ROS	Preclinical/Pilot clinical studies	[74,89,90]
Nanoparticle-based delivery systems	Enhance CNS penetration and bioavailability of therapeutic compounds	Preclinical/Translational research	[12,91,92,93]
Proteolysis-targeting chimeras (PROTACs)	Induce selective degradation of pathogenic proteins (e.g., tau, Aβ-related targets)	Preclinical concept/Early-stage development	[75,94,95,96]
Gene therapies	Deliver protective or restorative genes via viral vectors (mainly AAV) to enhance neuroprotection, modulate amyloid/tau pathology, and improve synaptic function	Phase 1/2 clinical trials; preliminary results show safety, biomarker improvements	[97,98,99]

**Table 4:** Selected antioxidant compounds and their potential role in AD.

Antioxidant Agent	Antioxidant Mechanism	Influence on AD	References
Resveratrol	Increases activity of antioxidant enzymes (SOD, GSH-Px, CAT) while reducing MDA levels	Neuroprotective effect, reduction in oxidative stress	[101]
Curcumin	Reduces MDA concentration, enhances total antioxidant capacity (TAC), scavenges reactive nitrogen and oxygen species, regulates enzymes and chelates metals, stabilizes ROS	Reduction in oxidative stress	[102,103]
Vitamin E	Scavenges free radicals, inhibits hydrogen peroxide production, improves performance in the water maze	Neuroprotection, reduced neuronal damage	[104,105]
Vitamin C	Counters ROS, regenerates antioxidant enzymes, scavenges oxygen and nitrogen radicals, donates hydrogen atoms to lipid radicals, quenches singlet oxygen, removes molecular oxygen, regenerates tocopherol from its oxidized form	Reduction in oxidative stress, enhanced neuronal protection	[106,107]
EGCG	Protects against t-butyl hydroperoxide, 6-hydroxydopamine, iron ions, UV radiation, hydrogen peroxide, 3-hydroxykynurenine; lowers TBARS, lipid hydroperoxides, 4-HNE, MDA; increases GPx and GSH activity	Protection against synaptic degeneration	[109,110]
Propolis	Scavenges free radicals, strong reducing capacity	Reduced neurotoxicity	[111]
Rutin	Binds Keap1 and blocks its interaction with Nrf2; increases activity of antioxidant enzymes (SOD, GPx, CAT)	Reduction in oxidative stress, decreased pro-inflammatory cytokines, inhibition of tau pathology, improved cognitive function in animal models	[112,113,114]
Sulforaphane	Activates Nrf2–ARE pathway, increases expression of SOD, CAT, GST; reduces HDAC1/3; enhances histone acetylation; epigenetic effects	Reduction in Aβ and tau, improved cognition, decreased oxidative stress, enhanced synaptic plasticity and blood–brain barrier integrity	[117,121,122]
Melatonin	Scavenges free radicals, activates Nrf2, stabilizes mitochondria, regulates PI3K/AKT/mTOR, SIRT1/FOXO, MAPK/ERK pathways	Reduction in Aβ and tau, improved sleep and mitochondrial function, circadian rhythm regulation, neurogenesis and cholinergic protection	[7,128,136]
NAC	Thiol group donor, GSH precursor, reduces 4-HNE, improves BBB integrity, stabilizes synapses, anti-inflammatory effect	Reduction in Aβ and phosphorylated tau, improved cognition, enhanced neurogenesis, glutathione support, synergistic effect with other antioxidants	[139,140,144]
Astaxanthin	Reduces ROS, activates Nrf2 pathway, lowers mTOR, stimulates autophagy, regulates miRNA-124, supports mitochondria	Improved memory, reduced Aβ and tau, anti-inflammatory and anti-apoptotic effects, enhanced neuroplasticity	[146,147,149]

**Table 5:** Comparative overview of major antioxidant strategies in Alzheimer’s disease therapy.

Compound/Approach	Molecular Targets/Mechanisms	Experimental Models	Key Outcomes	Limitations/Translational Issues
Resveratrol	↓ ROS, ↑ SIRT1, modulation of Aβ and tau, anti-inflammatory	in vitro, in vivo, early clinical trials	Neuroprotection, memory improvement, activation of antioxidant pathways	Low bioavailability, rapid metabolism
Curcumin	Antioxidant, anti-inflammatory, anti-amyloid	in vitro, in vivo, small clinical trials	Reduction in Aβ, improved mitochondrial function	Very low bioavailability, inconsistent clinical outcomes
Rutin	ROS scavenging, mitochondrial stabilization, anti-inflammatory	in vitro, in vivo	Memory improvement, reduced oxidative stress	No clinical data, poor solubility
Vitamins C and E	Classical antioxidants (ROS scavenging)	Animal models, clinical trials	Protective effects in models, mixed clinical results	Inconsistent clinical efficacy, limited effectiveness as monotherapy
Melatonin	Antioxidant, circadian rhythm regulation, mitochondrial modulation	in vitro, in vivo, clinical studies	Neuroprotection, improved sleep and memory	Variable dosing and bioavailability, inconclusive clinical evidence
Selenium and SeNPs	Support of selenoproteins (GPx, TrxR), ↓ ROS, inhibition of Aβ and tau, immunomodulation	in vitro, in vivo	Ebselen, SeNPs, and SELENOW improve cognition, reduce tau pathology	Mainly preclinical data, potential toxicity, lack of large clinical trials
MAO-B inhibitors (selegiline, rasagiline, ladostigil)	Reduction in ROS via MAO-B inhibition, neuroprotective effects	Animal models, clinical use	Cognitive improvement, indirect antioxidant effects	Established drugs, significant side effects and interactions
Novel approaches (nanoparticles, immunotherapy, mitochondrial/gene therapy)	Targeted drug delivery, redox pathway modulation, immunomodulation	in vitro, in vivo, preliminary clinical studies	Improved BBB penetration, synergistic effects, innovative strategies	Early-stage research, lack of large-scale clinical validation
